# Morphological and Chemical Profile of Three Tomato (*Solanum lycopersicum* L.) Landraces of A Semi-Arid Mediterranean Environment

**DOI:** 10.3390/plants8080273

**Published:** 2019-08-08

**Authors:** Massimiliano Renna, Massimiliano D’Imperio, Maria Gonnella, Miriana Durante, Angelo Parente, Giovanni Mita, Pietro Santamaria, Francesco Serio

**Affiliations:** 1 Institute of Sciences of Food Production, CNR—National Research Council of Italy, Via Amendola 122/O, 70126 Bari, Italy; 2 Institute of Sciences of Food Production (ISPA), CNR, via Lecce-Monteroni, 73100 Lecce, Italy; 3 Department of Agricultural and Environmental Science, University of Bari Aldo Moro, via Amendola 165/A, 70126 Bari, Italy

**Keywords:** agrobiodiversity, ascorbic acid, carotenoids, color, distinctiveness, local varieties, tocopherols, total phenols, total soluble solids

## Abstract

Puglia (Southern Italy), particularly rich in tomato agro-biodiversity, can be considered a typical region of the semi-arid Mediterranean environments. In this study, three local varieties of tomato (Manduria, Giallo di Crispiano and Regina) were characterized by using morphological descriptors according to international standards. Chemical (isoprenoids, ascorbic acid, total phenols, sugars and mineral content) and agronomic assessment were carried out to highlight the specific traits of these local varieties well adapted to a semi-arid environment. Data of morphological traits according to the “International Union for the Protection of New Varieties of Plants” (UPOV) guideline evidenced a clear distinctness among all three landraces, especially as regards fruits. Results also highlighted that a great part of variation in chemical traits was almost exclusively due to genotypes, while in a few cases observed differences resulted from the interaction between genotype and harvest time. The results of the present study may represent the first step toward the recognition of “conservation variety” status for Regina, Giallo di Crispiano and Manduria tomato landraces. At the same time, both quality traits and agronomic performance of these tomato genotypes suggest the possibility of their cultivation in other semi-arid environments also considering their quality traits, in view of a sustainable production.

## 1. Introduction

The Mediterranean traditional food products are greatly appreciated for both their high nutritional and sensorial value, also due to natural biodiversity of raw materials, and on traditional know how in food processing [1,2]. 

The plant biodiversity of the Mediterranean Basin is outstanding considering that it comprises about 25,000 plant species [3]. At the same time, it is estimated a 75% loss in the agro-biodiversity with respect to 1900s, mainly because of the wide use of high-yielding modern varieties [4]. Such loss of agro-biodiversity regards essentially the local varieties also known as landraces, farmer’s varieties, folk varieties. A local variety is a variable population, usually known with a local name, not already involved into a specific program of genetic improvement; characterized by a marked adaptation to a specific environmental and cultural system and closely associated with the uses, knowledge, habits and dialects of the local population that has developed and fosters its cultivation [5]. It is well known that the prevailing use of modern varieties has some advantages (e.g., a greater yield); nevertheless, it should be considered that for some vegetables, such as tomato (*Solanum lycopersicum* L.), some studies reported that various modern commercial varieties contain lower levels of important flavor compounds in comparison to the older local ones [5]. It is not by chance that an increased interest to promote traditional tomato landraces seems to be fueled by the need to preserve their desirable organoleptic quality traits [6]. Indeed, as Donald Davis states “breeding and intensified cultivation for high yield tend to reduce the concentration of nutrients” [7]. In this context, the local varieties of vegetables may represent an agronomical valid choice due to their high nutritional value, their lower water requirement as well as good adaptation to pests and poor soils [8]. Therefore, the cultivation of tomato landraces together with the application of specific crop-management techniques may represent a useful strategy of adaptation to warmer climate with a trend to drought [9].

An effective valorization of Mediterranean plant varieties, as sources of traditional food products with high nutritional and sensorial value, requires their unequivocal characterization, aiming to define their specific traits. Unfortunately, although many initiatives aiming to collect information about plants and the derived foods are currently going on, a comprehensive repository about the Mediterranean plant varieties is still lacking.

Puglia region (Southern Italy) is particularly rich in agro-biodiversity, representing an example of how vegetable landraces, largely used and requested by people, can still play an important role in the modern horticulture and contribute to build a complex food system, in which local culture and traditions are interconnected with both environment and local productions [5]. At the same time, the agro-biodiversity of vegetable crops in this region has been eroded in the last decades due to several factors such as ageing of the farming population and consequent farmland abandonment as well as loss of knowledge and historical memory, failure of information transfer between generations [10]. For these reasons the Puglia Region Administration launched two actions related to biodiversity under the 2007–2013 and 2014–2020 Rural Development Program (RDP) with the aim to preserve plant resources for example by promoting the exchange of information between stakeholders and facilitate the diffusion of knowledge. In this context, thanks to the project “Biodiversity of the Puglia’s vegetable crops (BiodiverSO: https://biodiversitapuglia.it//)”, a total of 141 genotypes were characterized for morphological, chemical and genetic traits [10].

Tomato is a species of great economic importance since it represents the second most cultivated vegetable worldwide [11,12], also for its richness in healthy compounds such as lycopene, vitamin A, ascorbic acid and tocopherols. Originating in the Andean regions, tomato has Italy as secondary centre of diversification, since a great number of landraces, with different fruit typologies, developed and diffused due to adaptation to both different environmental and cultivation conditions [6].

Puglia can be considered a region with a particular richness in tomato agro-biodiversity, since at least 27 landraces are known and described by a local variety name [10]. Among these, three local varieties, “Regina”, “Giallo di Crispiano” and “Manduria” [10], studied within the BiodiverSO project, could be of great interest with regard to morphological, qualitative and nutritional traits as well as from the ethnobotanical point of view. They show a high tolerance to drought too. All the three local varieties are registered in the ‘List of Traditional Agri-Food Products’ of the Ministry of Agricultural, Food and Forestry Policies. They are also classified as ‘Slow Food Presidium’ by the Slow Food Foundation [13], whose aim is to preserve authenticity and promote the origin of the Italian traditional food products.

Regina and Giallo di Crispiano are classified as long-storage tomatoes [14] and traditionally braided in bunches by tying the peduncles together with a cotton thread. So, a peculiarity of these landraces is that their fruits can be naturally preserved for several months after the summer harvest, ensuring availability of tomatoes during the winter period. Moreover, these landraces are highly appreciated by consumers for their peculiar taste and texture, which appear different from the commercial cultivars of cherry tomato. Manduria can be classified as a processing tomato type, since it is harvested at total ripened stage and destined mainly to the tomato processing chain, especially for sauces or canned production. This landrace shows interesting organoleptic traits as well as the ability to confer resistance to the infection of a Sw5-Breaking Strain of Tomato spotted wilt virus when used as a rootstock for grafting commercial cultivars of tomato [15].

Apart its richness in agro-biodiversity and the peculiarity of some local varieties, it is important to highlight that Puglia, placed in the center of the Mediterranean Basin, can be considered a typical region of the semi-arid Mediterranean environments with hot and dry summer and mild and rainy winter season [16]; temperatures may exceed 40 °C in summer, while annual rainfall ranges between 400 and 550 mm [10]. The dominant soils are Cambisols, Luvisols and Vertisols, characterised by cretaceous limestone, marl and clayey to sandy deposits [16].

Considering all the above remarks and the lacking of a comprehensive knowledge about the local vegetable varieties, the aims of the present study were: (i) To carry out a morphological characterization by using both descriptors and scores according to international standards; (ii) to evaluate and compare some chemical, agronomic and morphological traits of tomato fruits from plants grown in the same semi-arid environment. The more general goal was to increase the knowledge about these examples of horticultural plants diversity, which can be exploited for cultivation and direct use in markets, breeding purposes as well as for future genetic/molecular investigations.

## 2. Results

### 2.1. Morphological Characterization

The descriptors listed in the UPOV (International Union for the Protection of new Varieties of Plants) Guideline TG/44/10 [17] were used to score the three landraces (Table 1). 

**Figure 1:**
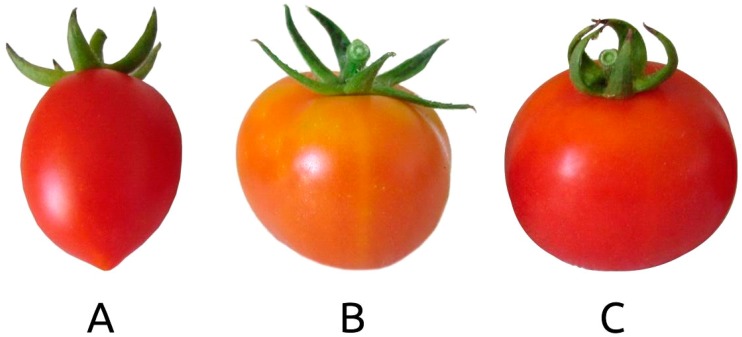
Fruits of the three tomato landraces harvested at commercial ripening: (**A**) Manduria; (**B**) Giallo di Crispiano; (**C**) Regina.

The three landraces showed the same determinate plant growth type and flower traits as well as time of flowering and maturity (Table 1). Inflorescence appearance started in the first decade of May for Regina and Manduria, a week later for Giallo di Crispiano. As far as leaf traits, no differences were found between the three landraces with the exception of division of blade (LfDB). Regarding tomato fruits, most morphological traits showed variation among the three landraces. For example, Manduria landrace showed a smaller fruit size (FrSz) than the other two landraces (Figure 1), while the score of ratio length/width (FrLW) was lowest in Regina tomato fruits. The shape in longitudinal section (FrSL) was rectangular in Manduria, circular in Giallo di Crispiano and slightly flattened in Regina (Table 1). Only two locules (FrNL) were found in fruits of both Manduria and Regina landraces, while Giallo di Crispiano landrace showed two or three locules in fruits. At maturity, fruits of both Manduria and Regina landraces showed a red color (flesh and external part—FrCF and FrC), while Giallo di Crispiano showed an orange color of both flesh and skin of fruits (Table 1; Figure 1). The score of firmness (FrF) was “medium” in Manduria and “firm” in both Giallo di Crispiano and Regina landraces. At the same time, in all three landraces the green shoulder was absent while the cross section was “not round” (Table 1).

### 2.2. Physical Analysis and Titratable Acidity

**Figure 2:**
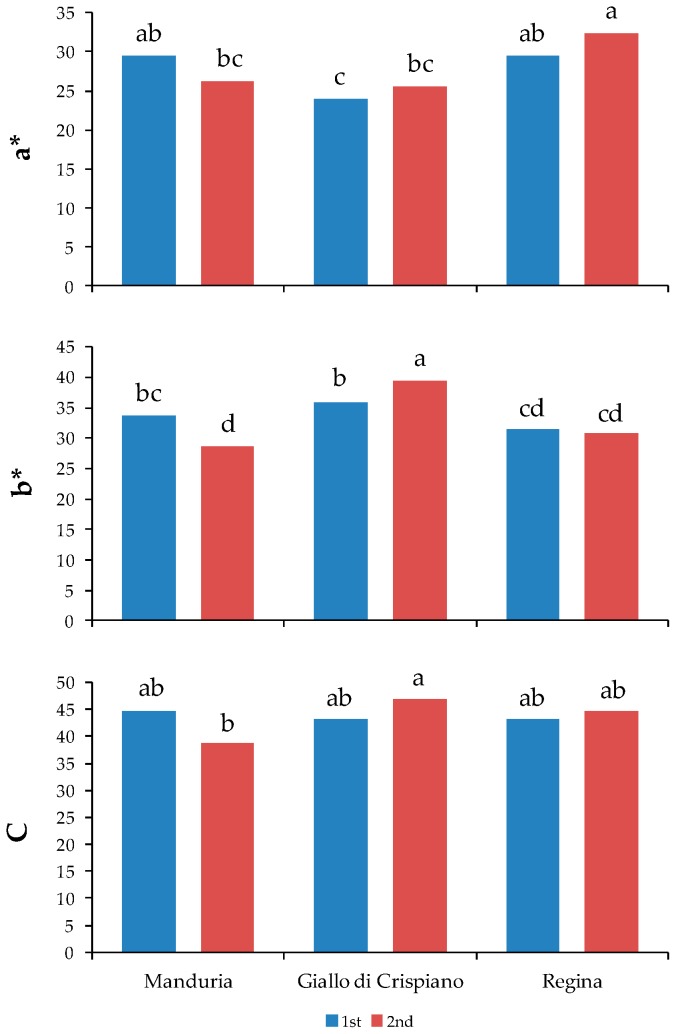
Color parameters (a*, b* and C) of tomato fruits of three landraces at two harvest time: (1st) 7th, 14th and 20th July 2016, respectively for Manduria, Regina and Giallo di Crispiano; (2nd) 14th and 20th July and 4th August 2016, respectively for Manduria, Regina and Giallo di Crispiano. Interaction significance for all parameters: *p* ≤ 0.05. Different letters indicate significant differences at *p* = 0.05.

The color parameters obtained by using a color-meter are reported in Table 2 and Figure 2.

Generally, Giallo di Crispiano landrace showed the highest lightness value and for all three landraces this parameter was higher at the 2nd harvest than at the 1st one (Table 2). Regarding hue angle, Giallo di Crispiano showed a higher value than Manduria and Regina landraces. The highest a* values were found in Manduria at the 1st harvest and in Regina at both harvests, while the highest b* value was found in Giallo di Crispiano at the 2nd harvest. Regarding *C* value, the unique real difference was between Manduria and Giallo di Crispiano, both at the 2nd harvest (Figure 2).

Regarding weight, diameters, dry matter, total soluble solids and titratable acidity of fruits, significant interactions between genotype and harvest were never found (Table 3).

Regina fruits showed the highest average weight and for all three landraces this parameter was higher at the 1st harvest than at the second one. Overall, the fruit polar diameter was highest in Manduria, while the equatorial diameter was highest in Regina. The average dry matter and total soluble solids (TSS) were, respectively, 8.72 g 100 g^−1^ fresh weight (FW) and 6.4 °Brix without any differences between the three genotypes and the two harvests. The highest value of titratable acidity (TA) was found in Manduria, while the highest TSS/TA ratio was found in Regina (Table 3).

### 2.3. Chemical Traits

Regina fruits showed the highest values of glucose, fructose and sweetness index, while total phenols content was highest in Giallo di Crispiano (Table 4). In general, total phenols content was higher at the 2nd harvest than at first (Table 4). Tomato fruits showed an average glucose/fructose ratio of 0.865 without any differences between the three genotypes and the harvest time (Table 4).

**Figure 3:**
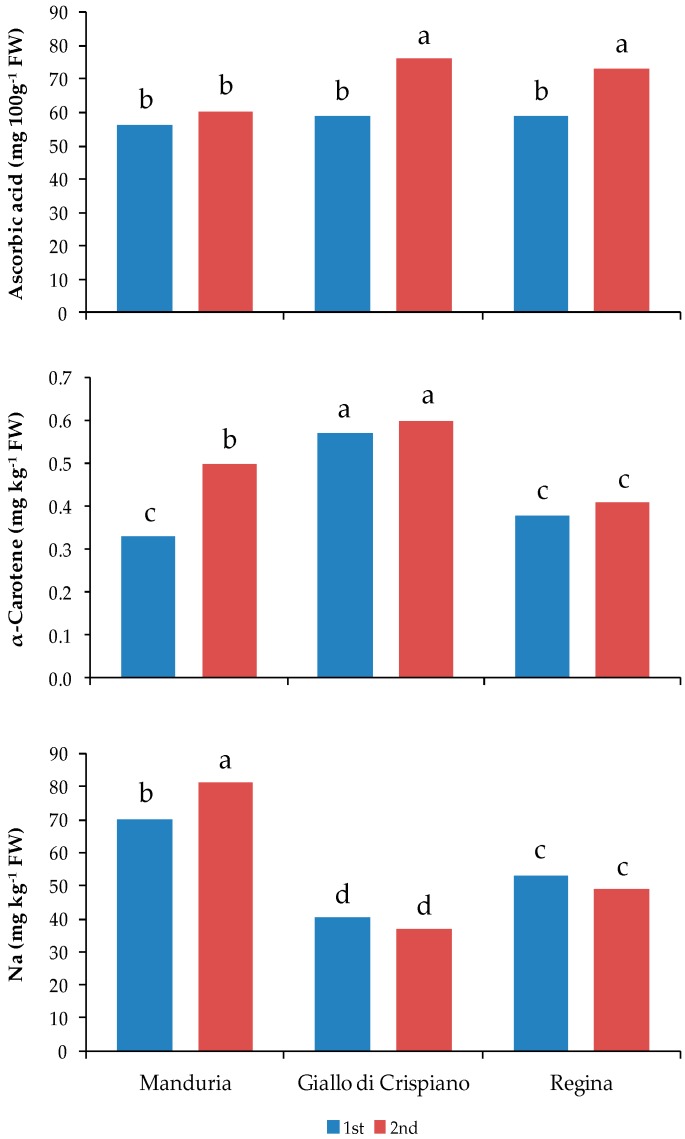
Content of ascorbic acid (*p* ≤ 0.01), α-Carotene (*p* ≤ 0.05) and sodium (*p* ≤ 0.05) in tomato fruits of three genotypes at two harvest time: (1st) 7th, 14th and 20th July 2016, respectively for Manduria, Regina and Giallo di Crispiano; (2nd) 14th and 20th July and 4th August 2016, respectively for Manduria, Regina and Giallo di Crispiano. Different letters indicate significant differences at *p* = 0.05.

A significant interaction between genotype and harvest was found only for the ascorbic acid (Table 4) and the highest values were found in fruits of both Giallo di Crispiano and Regina at the 2nd harvest (Figure 3).

The highest values of α-Carotene were found in fruits of Giallo di Crispiano, for both harvests, while only for Manduria were found differences between the two harvests (Figure 3). As far as other carotenoids and tocopherols, the interaction between genotype and harvest was not significant (Table 5).

For all three landraces the average contents of lutein, lycopene and β + γ tocopherol were, respectively, 3.43, 18.24 and 2.33 mg kg^−1^ FW, independently on the genotype and harvest (Table 5), while the highest content of β-Carotene and α-tocopherol was showed by Giallo di Crispiano (Table 5).

The highest value of sodium was found in fruits of Manduria at the 2nd harvest, while the lowest value was found in Giallo di Crispiano regardless of the harvest time (Figure 3).

For all three landraces the average contents of potassium, boron and copper were, respectively, 3026, 1.06 and 5.63 mg kg^−1^ FW, independently of the genotype and harvest (Table 6). Manduria and Giallo di Crispiano showed a higher manganese content (0.62 mg kg^−1^ FW, on average) than Regina, while Giallo di Crispiano showed the highest content of zinc (Table 6). Generally, the content of calcium and magnesium was higher at the 2nd harvest than at the first, while the average iron content was higher at the 1st one (Table 6).

### 2.4. Principal Component Analysis

**Figure 4:**
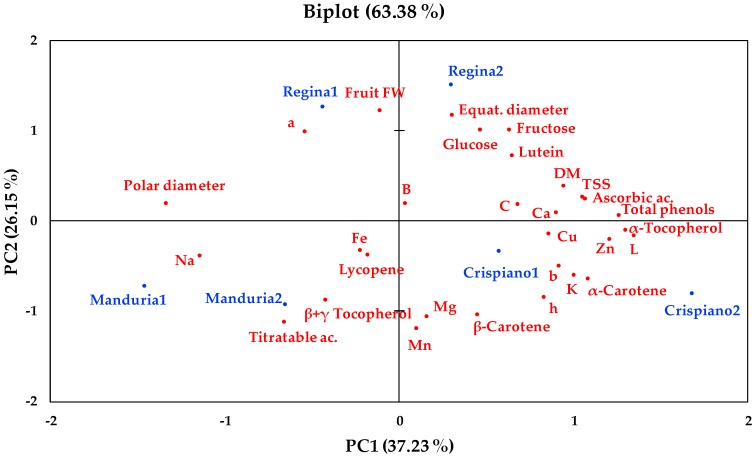
PCA biplot (Principal Component 1 - PC1 *vs* Principal Component 2 -PC2) describing the variation of the physical and chemical parameters used to characterize the three studied local tomato landraces. For each landrace, numbers 1 and 2 indicate the first and the second harvest, respectively. DM, dry matter; FW, fresh weight; L, lightness; C, color saturation; h, hue angle; a, red/green chromaticity; b, yellow/blues chromaticity; B, boron; Ca, calcium; Cu, cupper; Zn, zinc; Mg, magnesium; Mn, manganese; Na, sodium, K, potassium; Fe, iron.

The principal component analysis (PCA) highlighted all the differences by the analysis of variance (ANOVA) results, giving a clear plot summary visualization. The eigenvalues of the correlation matrix resulted in the first three Principal Components (PCs) explaining 79% of the total variance. In the PCA biplot (Figure 4) the first two PCs explained 37.23% and 26.15%, respectively.

The PCA allowed to separate distinctly Regina from the other two landraces, since they are collocated on the two opposite sides of the PC2. Fruit weight and equatorial diameter together with glucose and fructose and a* parameter resulted positively correlated with PC2; while Mg and Mn, titratable acidity and β-Carotene were negatively correlated with PC2. Actually, Regina showed the highest values of the first group of parameters and the lowest values of the second group, compared to the other landraces (Table 2, Table 3, Table 4, Table 5 and Table 6). In addition, Manduria and Giallo di Crispiano were associated with the negative and positive sides of PC1, respectively. L* parameter, α- tocopherol, total phenols were highly positively correlated with PC1, together with, α-Carotene, ascorbic acid, TSS, Zn and K (Figure 4). At the same time, Na and fruit polar diameter were highly correlated with the negative portion of PC1, influencing Manduria landrace (Figure 4).

## 3. Discussion

In this study, the morphological characterization, by using UPOV descriptors was carried out, to realize a preliminary repository aimed to encourage the exploitation of these local varieties for direct use by farmers as well as for breeding purpose. It must be highlighted that, especially in the case of cultivation, the diffusion of plant genotypes requires their registration in a varietal register. At the same time, it should be considered that in Italy a great number of local varieties is lacking in the common catalogue of varieties, essentially due to the absence of commercial interest from the specialized seed companies [6]. Therefore, the European Union has established a specific section of the varietal register regarding the “conservation varieties” [5], a new type of agricultural varieties that can be marketed in Europe for both preserving the plant genetic resources and favoring the marketing of their seeds [18].

According to the Commission Directive 2009/145/EC [19], the distinctness of varieties for entering into the common catalogue of varieties as a “conservation variety” can be ensured by using UPOV descriptors. In this context the results of the present study may represent the first step toward the recognition of “conservation variety” status for Regina, Giallo di Crispiano and Manduria tomato landraces.

Data on morphological traits scored in Regina, Giallo di Crispiano and Manduria according to the UPOV guideline evidenced a clear distinctness among these local varieties, especially as regards some fruit traits, such as size, ratio length/width, shape in longitudinal section, number of locules and color at maturity (Table 1). Moreover, a wide characterization of chemical, agronomic and morphological traits may be useful in order to evaluate and distinguish local varieties also for their quality and nutritional traits. In this way, the choice to cultivate one of these varieties could be due to some of these peculiarities. Finally, a wide characterization may be a useful tool in the hands of breeders for improving the traditional varieties without losing those peculiar traits even in a modern agriculture scenario. Therefore, in this work we pursued also the analysis of other several traits including chemical and physical ones.

Our analysis highlighted that a great part of variation in both physical and chemical traits was almost exclusively due to genotypes, while only in a few cases we found differences due to the interaction between genotype and harvest time.

Regina tomato differs from the other landraces for fruits with higher weight and equatorial diameter (Table 3). Moreover, Regina tomato showed a higher content of sugars since glucose level resulted 12% higher than in the other landraces and the fructose content was 5% and 15% higher than in Giallo di Crispiano and Manduria, respectively (Table 4). As expected, the highest sugar content in Regina fruits also results in a higher sweetness index that is also an indicator of consumer acceptance [20]. Thus, considering the preference of consumers for sweet tomatoes it is possible that the highest sweetness of Regina tomato may positively influence consumers’ choice and acceptance. In a previous study, Barbagallo et al. [21] analyzed the sugar content of tomato in six local genotypes of cherry tomato from Sicily and reported a glucose content ranging between 1440 and 2150 mg 100 g^−1^ FW and a fructose content ranging between 1470 and 2200 g 100 g^−1^ FW. Moreover, in the widespread commercial cultivar Naomi, glucose and fructose content ranging between 1590 and 2500 g 100 g^−1^ FW, 1640 and 2700 g 100 g^−1^ FW, respectively [21,22]. Therefore, the results of our study indicate that, as far as the sugar content, especially Regina landrace is quite interesting and it is comparable with a commercial cultivar.

Titratable acidity (TA) was a distinctive trait for all the three landraces, while no differences were found as regards total soluble solids (TSS). Thus, also the ratio TSS/TA was directly influenced by the TA (Table 3). TA is an important quality attribute for processing tomatoes, because the higher this value, the easier it is to control microbial deteriorations in processed tomato products such as canned products [23]. TA in tomato fruits can be affected by several factors [24] however the average acidity value of processing tomatoes is 0.35 g 100 g^−1^ FW [23]. Considering that Manduria is destined mainly to the processing chain, our results confirm its good aptitude as processing tomato, since it shows the highest TA value among the three landraces (Table 4).

As regard to the content of the nutritional quality-related compounds, both ANOVA and PCA analyses show that Giallo di Crispiano differs from the other landraces for its higher content of total phenols, α-tocopherol, α- and β-Carotene (Table 4 and Table 5, Figure 3 and Figure 4). α-Tocopherol is the most important isoform of the vitamin E, considering that it represents more than 90% respect to the total tocopherol isoforms and that it shows the highest biological activity when compared to the other isoforms, being absorbed preferentially by the human body [25]. In a study aimed to evaluate the content of both carotenoids and antioxidants of 12 tomato genotypes for fresh consumption (salad tomatoes) and 15 processing cultivars, some authors [26] reported a content of α-tocopherol ranging from 1.23 to 6.12 mg kg^−1^ FW in salad tomatoes and 4.11 to 11.64 mg kg^−1^ FW in processing cultivars. Our results are in agreement with these authors, confirming that α-tocopherol content in tomato fruits is mainly influenced by genotypes. Moreover, it is worthwhile noting that in the three studied landraces a higher α-tocopherol content than that reported on tomato F1 hybrids [27] was observed. From a nutritional point of view, it should be considered that the vitamin E recommendations refer to α-tocopherol alone, the only form maintained in plasma [28]. In this context, it is interesting to note that especially Giallo di Crispiano could be considered a very good source of this antioxidant important for human health, since it shows a α-tocopherol content significantly higher respect to other tomato genotypes.

**Figure 5:**
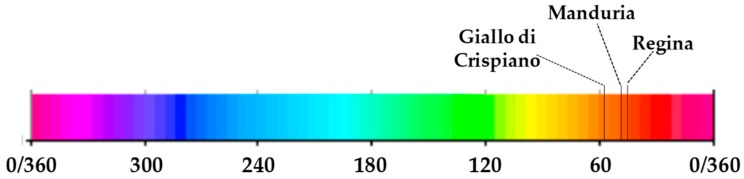
Graphical representation of hue angle (CIE *Lab*) detected for the three tomato landraces.

Carotenoids are lipophilic pigments, providing orange, yellow, red and purple colors in fruits and vegetables, that are known to possess a good antioxidant activity against free radicals [26]. β-Carotene is one of the predominant carotenoids in tomato and represents an important compound for human health, being the precursor of the vitamin A [25,26]. The content of β-Carotene in tomatoes is 3–6 mg kg^−1^ FW in salad tomatoes and 2–4.5 mg kg^−1^ FW in processing cultivars [26]. Our results show that all three landraces can be considered as a good source of β-Carotene. Moreover, it is interesting to note that the highest β-Carotene content observed in Giallo di Crispiano, not only positively influences its nutritional profile, but it is also important to increase the visual quality trait such as color. Indeed, color is one of the most important quality attribute of fruits and vegetables, influencing consumers’ choice and preference [29]. Regarding tomato, it is well known that the wide range of red color in ripe fruits is mainly due to the synthesis of lycopene and β-Carotene in different proportion. Since β-Carotene pigment is responsible for the orange color in fruits and vegetables [29] and considering that lycopene content did not differ among the three genotypes (Table 5), our results suggest that the higher β-Carotene content in Giallo di Crispiano might be the reason why it is perceived as less “red” than the other landraces. This is confirmed by CIE *Lab* color analysis, considering that hue angle value was higher in Giallo di Crispiano than in the other landraces (Table 2). Hue angle is considered the “qualitative” attribute of color, according to which colors can be traditionally defined as reddish, greenish, etc. [29]. A hue angle value of 0 or 360 represents red hue, whilst hue angle values of 90, 180 and 270 represent yellow, green and blue hues, respectively. Thus, on the basis of the graphical representation of hue angle for the three tomato landraces (Figure 5) it is possible to better understand that Giallo di Crispiano landrace can be considered less “red” than Manduria and Regina.

It is no by chance that the term “giallo” (Italian for yellow) is a part of the name of this local variety. Anyway, the characteristic color of Giallo di Crispiano tomatoes could be an interesting and peculiar trait to be introduced into a modern background, considering that advances in plant breeding have resulted in new cultivars with a wide range of colors such as the yellow tomatoes [29] (i.e., Lemonade^TM^, Yolita^TM^, ext.) [30].

Phenolics and isoprenoids are other important bioactive compounds in tomato. Indeed, tomato fruits can contain a great amount of several phenolics, well known for their antioxidant capacity and other properties, such as anti-inflammatory, cardioprotective, anti-microbial, and neuroprotective effects [6]. Phenolic compounds can be considered as very efficient scavengers of peroxyl radicals due to their peculiar chemical structure. Furthermore, it should be considered their capacity to reduce and chelate ferric iron that is involved in lipid peroxidation [31]. In a study aimed to evaluate nine commercial varieties of tomato, Martìnez-Valverde et al. [31] observed a content of total phenols ranging between 26 and 50 mg 100 g^−1^ FW, while Baldina et al. [6] analysing eighteen genotypes of tomatoes reported phenol levels lower than 10 mg 100 g^−1^ FW. Figàs et al. [32] showed a total phenols content ranging between 49 and 117 mg 100 g^−1^ FW in sixty-nine tomato accessions from eight cultivar groups. The content of phenolic compounds in tomatoes can be affected by several factors such as genotype, availability of nitrogen in the root zone, biotic and abiotic stress-related events [25]. Our results confirm the great influence of genotype and highlight the high content of these important antioxidant compounds in all the three studied local varieties. At the same time, it is important to highlight that the phenol content in tomatoes can positively influence organoleptic traits [32]. Therefore, starting from these local varieties, a possible selection and/or breeding activity could be oriented also considering the phenol content in tomato fruits for both functional and taste quality.

In our study the ascorbic acid content was one of the few functional traits affected by the interaction between genotype and harvest, with the highest values found at the 2nd harvest in both Giallo di Crispiano and Regina (on average 74.6 mg 100 g^−1^ FW: Figure 3). It is well known that the ascorbic acid content is influenced by genotypes, however several environmental factors, such as growing environment, canopy, salinity of growing media, harvest time as well as season and weather trend within the same growing period, may affect the content of this vitamin in tomato fruits [25,33,34]. Table 7 shows the weather trend (temperatures and rains) from transplanting to harvest for all the three local varieties.

Both Regina and Giallo di Crispiano the second harvest was carried out after some days of rain and lower temperatures (Table 7: 10th decade after transplanting). These different weather conditions, respect to ones of the second harvest of Manduria (carried out within the 9th decade after transplanting, before the rainfall), could have influenced the tomato plants physiology also considering the increased water availability after the rainy event. It is interesting to underline that an optimal water availability for plants can induce a good availability of calcium, which plays a positive role in the biosynthesis of ascorbic acid [25]. This is in agreement with the higher average calcium content in tomato fruits at the second harvest (Table 6), especially in Giallo di Crispiano (data not shown) and the strong positive correlation (Pearson correlation coefficients = 0.83) between ascorbic acid and calcium contents (Table S1). Therefore, considering all the above remark, the results of the present study confirm that different contents of ascorbic acid in tomato fruits depend on both the genotypes and several other factors. At the same time, it is interesting to note that all three landraces can be considered a very good source of ascorbic acid (about 64 mg 100 g^−1^ FW, on average).

Sodium content in tomato fruits was the only mineral element affected by the interaction between genotype and harvest. The highest sodium content was found in Manduria at the 2nd harvest, followed by the same genotype at the 1st one (Figure 3). On average, the Na content in Manduria fruits was about 49% and 96% higher than values found in Regina and Giallo di Crispiano, respectively. Anyway, as reported by the European Food Safety Authority, the daily sodium needs are 1500 mg, which roughly corresponds to the mean recommended amount as daily maximum intake by the Scientific Committee on Food (1993) and by several international recommendations [36]. Given these values, the differences found in our study between the three landraces could be considered negligible from a nutritional point of view. At the same time, from a plant physiological point of view, it should be considered that different sodium content in tomato fruits can be due to a different sodium partitioning within the shoot [37]. Therefore, our results suggest that: (i) Sodium content in tomato fruits was influenced by genotypes; (ii) Giallo di Crispiano may be considered an interesting landrace in the case of growing under high salinity conditions. For all the other minerals no remarkable differences can be appreciated with the exception of Mn and Zn. In this context it is important to highlight that Giallo di Crispiano shows a zinc content (4.22 mg kg^−1^ FW) about 60% and 124% higher than Regina and Manduria, respectively. Ordóñez-Santos et al. [38] found a zinc concentration between 1.4 and 3.3 mg kg^−1^ FW in organic and conventional tomatoes, while the National Nutrient Database of the United States Department of Agriculture indicated an average zinc content of 1.7, 1.4 and 2.8 mg kg^−1^ FW respectively for red, orange and yellow tomatoes [39]. Our results suggest that Giallo di Crispiano could be considered a very good source of this important element for human health, considering that zinc plays a key role in several function of the human body [40].

Apart from the morphological and chemical profile, from an agronomic point of view it could be interesting to highlight the yield performance of each landrace, in the perspective of increasing the overall knowledge and possible exploitation of the local varieties. In this context it is important to highlight that in literature very few studies on tomato characterization report also agronomic information, giving a wide description of morphological, chemical, physical and agronomic features, as for long-storage tomato landraces in this case. In our study, the overall yield was about 886, 818, and 551 g tomatoes plant^−1^ corresponding to a 29.5, 26.3 and 18.4 t ha^−1^, respectively for Manduria, Regina and Giallo di Crispiano. Crop performance of the studied local varieties can be evaluated as satisfactory, considering: (i) Plants habit (determinate growth); (ii) small size of fruits (like cherry tomatoes); (iii) low required technical input (especially water). Regarding this last point it is important to note that during the growing cycle the cumulated rainfall was about 150 mm (Table 7) and only a total of 50 mm of water was distributed as supplemental irrigation between flowering and fruit growth. Therefore, all three landraces could be considered as well-adapted genotypes to a semi-arid environment and suitable to be potentially chosen for cultivation not only for their quality traits but also in terms of a sustainable production.

## 4. Materials and Methods

### 4.1. Cropping Details

Three tomato landraces were cultivated in open field at “La Noria” experimental farm of the Institute of Sciences of Food Production of the Italian National Research Council, located in Mola di Bari (Bari, Italy), 24 m above sea level; 41°03′ N, 17°04′ E. Seedlings of Regina, di Manduria and Giallo di Crispiano were provided by Vivaio F.lli Corrado (Brindisi, Italy) and Vivaio Ortovivaistica (Francavilla Fontana, Brindisi, Italy) using self-produced and selected seeds of the three landraces. Transplant was carried out at the four-leaf stage on 14 April 2016, at 100 cm between rows and 30 cm within row (3.3 plants m^−2^). A randomized block design with three replications was adopted in the field. Plants were grown according to the agricultural practices of farmers specialized in the cultivation of local tomato landraces. The experimental field was on clay soil under Mediterranean climate, with the summer season characterized by scarce rain and maximum temperatures even higher to 30–35 °C in the hottest period. For this reason, a drip irrigation system was set up to carry out supplemental irrigation at transplanting and during the growing cycle. At transplant, 100–80–100 kg ha^−1^ of N–P_2_O_5_–K_2_O fertilizers were applied. The harvest was performed according to the progressive ripening of fruits, following the criterion that the fruits were completely colored for more than 90%. Two sampling areas were delimited for each field replication. In each area two consecutive harvests were carried out in order to calculate the total cumulated yield.

### 4.2. Morphological Characterization

To assess morphological traits of the three landraces, 37 traits were scored by using descriptors detailed in Table 1, according to the UPOV (International Union for the Protection of new Varieties of Plants) Guideline TG/44/10 for the conduct of tests for Distinctness, Uniformity and Stability on tomato (UPOV Species Code: LYCOP_ESC), on 15 plants per genotype for a total of 45 plants, considering the three experimental replications.

### 4.3. Color Analysis, TSS Content and TA

CIELAB color parameters (L*, a*, b*) were detected by using the colorimeter CR-400 Chroma Meter (Minolta Co., Osaka, Japan). Each measurement was carried out in triplicate on fruits skin at five random point. Starting from L* (lightness), a* (red/green attribute) and b* (yellow/blue) parameters, also C (saturation) and h° (hue angle) were calculated by following functions: (i) C = [(a*)^2^ + (b*)^2^]^1⁄2^; (ii) h° = tan^−1^ (b*/a*). TSS content was measured using the refractometer DBR35 (XS Instruments, Italy) on a liquid fruits extract obtained by homogenizing 100 g of sample in a blender (Sterilmimex lab, International PBI, Milan, Italy), and then filtering the juice; results were expressed in °Brix. TA was measured using a Technotrate Digital Burette (Kartell, Milan, Italy) by potentiometric titration of fruits juice (10 mL) to pH 8.1 using NaOH (0.1 M) in the presence of phenolphthalein; results were expressed as percentage of citric acid equivalents in the juice.

### 4.4. Samples Preparation for Chemical Analysis

Before chemical analyses tomato fruits samples from each replication were divided into two equal portions. One portion was oven-dried in forced air at 105 °C until constant mass, for the determination of the dry weight (DW) content. Results were expressed as g 100 g^−1^ fresh weight (FW). The other portion was lyophilized by a LABCONCO FreeZone^®^ Freeze Dry System, model 7754030, (Kansas City, MI, USA) equipped with a LABCONCO FreeZone^®^ Stoppering Tray Dryer, model 7948030 (Kansas City, MI, USA). The freeze-dried matter was ground at 500 μm by using a Retsch laboratory mill (Torre Boldone, BG, Italy) to obtain a homogeneous powder.

### 4.5. Isoprenoids Extraction and Determination

Isoprenoids (carotenoids and tocopherols) were extracted from triplicate aliquots of freeze dried tomato powder (0.1 g) as reported by Sadler et al. [41] modified by Perkins-Veazie et al. [42]. The extracts were assayed as described by Fraser et al. [43] using an 1100 Series HPLC system (Agilent Technologies, California, USA) equipped with a YMC Reversed-Phase HPLC C30 Columns (Wilmington, NC, USA). The chromatographic separation was performed using the following solvents: methanol (A), water/methanol (20:80 *v/v*) containing 0.2% ammonium acetate (B), and tert-methyl butyl ether (C). Samples were initially eluted with 95% A and 5% B; 0 to 12 min, 80% A, 5% B, and 15% C; 12 to 42 min, 30% A, 5% B, and 65% C; 42 to 60 min, 30% A, 5% B, and 65% C; 60 to 62 min, 95% A, and 5% B. Ten microliter aliquots of the samples were injected in the HPLC-DAD system. The flow rate was 1.0 mL min^−1^, and the column temperature was maintained at 25 °C. Diode array detection was performed at 475 nm for carotenoids and 290 nm for tocopherols. Peaks were identified by comparing their retention times and UV-vis spectra to those of authentic standards. α-tocopherol, ß-tocopherol and γ-tocopherol were purchased from Cayman chemicals (Ann Arbor, MI, USA). Lutein, α-Carotene, ß-Carotene and lycopene were purchased from CaroteNature (Lupsingen, Base-land, Switzerland).

### 4.6. Ascorbic Acid and Total Phenols Determination

Ascorbic acid was extracted from triplicate aliquots of freeze dried tomato powder as reported by by Ferreira et al. [44] with some modifications. Briefly, 0.1 g of samples were extracted by 10 mL of 1% (w/v) metaphosphoric acid followed by shaking for 45 min at room temperature. Then, samples were centrifuged at 4000 *g* for 10 min. Supernatant (1 mL) was mixed with 9 mL of 0.005% 2,6-dichlorophenolindophenol (DCPIP) and the absorbance was measured within 30 min at 515 nm against a blank. To determine ascorbic acid content, a calibration curve of authentic L-ascorbic acid was used.

For total phenols the extraction procedure reported by Luthria et al. [45] with some modifications was used. The total polyphenols (TP) content in tomato sample was determined by using the Folin Ciocalteu method. Briefly, approximately 0.2 g of lyophilized sample were mixed with solvent mixture (MeOH:H_2_O:CH_3_COOH 79:20:1% *v/v/v*). The vials were then placed in a sonicator bath at ambient temperature for 30 min, followed by 1 h in magnetic stirred. The sample was centrifuged at 10,000× *g* for 10 min at 4 °C and the supernatant was transferred into a volumetric tube. The residue was resuspended in fresh solvent mixture, gently mixed manually and sonicated for 30 min followed stirring (1 h) and centrifugation (10,000× *g* for 10 min at 4 °C). The supernatant obtained was combined with the initial extract and appropriate aliquots of extracts were filtered (0.45 µm) and assayed for TP. The content of TP was determined using gallic acid (*R^2^* = 0.9922) as a calibration standard by using a Perkin-Elmer Lambda 25 spectrophotometer (Boston, MA, USA).

### 4.7. Glucose and Fructose Assay, and Sweetness Index

Triplicate aliquots of freeze-dried tomato powder were used to determine glucose and fructose contents, by ionic chromatography (Dionex model DX500; Dionex Corp., Sunnyvale, CA) using a pulsed amperometric detector (PAD) according to protocols used by Gonnella et al. [46]. Peak separation was performed using a Dionex CarboPac PA1 and isocratic elution with 50 mmol L^−1^ NaOH. Results were expressed as mg g^−1^ FW.

The sweetness index (SI) was calculated based on content and sweetness properties of individual carbohydrates by multiplying the sweetness coefficient of each sugar (glucose = 1.00 and fructose = 2.30) by the concentration (g 100 g^−1^ FW) of that sugar in fruits [47].

### 4.8. Elemental Analysis

Macro and microelements, Ca, K, Mg, Na, B, Mn, Zn, Fe and Cu, concentrations were determined according to D’Imperio et al. [48]. Briefly, a representative amount, 0.25 g, of freeze-dried tomato powder samples was digested in a closed-vessel microwave digestion system (MARS 6, CEM Corporation, Matthews, NC, USA) with 65% HNO_3_ (10 mL). The microwave digestion protocol was applied in two steps: 15 min up to 200 °C and 10 min at 200 °C stable (power set at 900–1050 W; 800 psi) by using HNO_3_ without sample as blanks. After mineralization the samples, diluted with ultrapure H_2_O (Milli-Q Millipore 18 M Ω/cm), were filtered using a 0.45 μm filter. The mineralized samples were analysed with the agilent 5100 Vertical Dual View ICP-OES (Santa Clara, CA, USA) to measure Ca, K, Mg and Na in radial mode and the minor elements (B, Mn, Zn, Fe and Cu) in axial mode.

### 4.9. Statistical Analysis

A two-way analysis of variance (ANOVA) was performed using the General Linear Model (GLM) procedure (SAS software, Version 9.1) applying a strip-plot design with harvest time and genotype as main factors. The separation of means was obtained by the Student–Newman–Keuls (SNK) test.

For a visual analysis of data, Principal Component Analysis (PCA) (PRINCOMP procedure, SAS software, Cary, NC, USA) was performed on mean centered and standardized (unit variance scaled) data prior to analysis. The data matrix submitted to PCA was made up of six observations (3 landraces and 2 harvests) and 30 variables (fruit FW, polar diameter, equatorial diameter, DM, TSS, titratable acidity, Na, Ca, Mg, K, B, Mn, Fe, Zn, Cu, lightness, a*, b*, chroma, hue angle, glucose, fructose, lutein, total phenols, ascorbic acid, α-tocopherol, β+γ-tocopherol, α-Carotene, β-Carotene, lycopene).

## 5. Conclusions

Puglia region (Southern Italy), with its high richness of biodiversity, can be considered as an example in which landraces vegetables can strongly interact with modern agriculture toward a complex food system, throughout an interconnection between environment, traditions and local productions. In this study, in three Apulian tomato landraces the morphological characterization as well as the quality and agronomic assessment were carried out in order to highlight the specific traits of these local varieties well adapted to a semi-arid environment. The results reported may represent the first step toward the recognition of “conservation variety” status for Regina, Giallo di Crispiano and Manduria tomato landraces. Moreover, our data reveal that all three local varieties are different for qualitative traits and, therefore, amenable to selection for breeding purpose. At the same time, both quality traits and agronomic performance of these tomato genotypes suggest the possibility of cultivation in other semi-arid environments also in consideration of a sustainable production. Finally, the exploitation of these local varieties may represent the next goal towards the diversification of the current food market and the valorization of the local agro-biodiversity.

## Figures and Tables

**Table 1:** Data of morphological traits scored in the three landraces according to the UPOV (International Union for the Protection of new Varieties of Plants) Guideline TG/44/10 for the conduct of tests for Distinctness, Uniformity and Stability on tomato (UPOV Species Code: LYCOP_ESC).

Descriptor	Acronym	Score Scale	Score		
			Manduria	Giallo di Crispiano	Regina
Plant: growth type	PGT	1, determinate; 2, indeterminate	1	1	1
Plant: number of inflorescences on main stem	PNI	3, few; 5, medium; 7, many	3	3	3
Stem: anthocyanin coloration of upper third	StAC	1, absent or very weak; 3, weak; 5, medium; 7, strong; 9, very strong	3	3	1
Leaf: length	LfL	3, short; 5, medium; 7, long	3	3	3
Leaf: width	LfW	3, narrow; 5, medium; 7, broad	3	3	3
Leaf: division of blade	LfDB	1, pinnate; 2, bipennate	1	2	1
Leaf: size of leaflets (in middle of leaf)	LfSL	1, very small; 3, small; 5, medium; 7, large; 9, very large	3	3	3
Leaf: intensity of green color	LfIG	3, light; 5, medium; 7, dark	3	3	3
Leaf: glossiness (in middle third of plant)	LfG	3, weak; 5, medium; 7, strong	3	3	3
Leaf: blistering (in middle third of plant)	LfB	3, weak; 5, medium; 7, strong	3	3	3
Leaf: size of blisters	LfSB	3, small; 5, medium; 7, large	3	3	3
Leaf: attitude of petiole of leaflet in relation to main axis (in middle third of plant)	LfAP	3, semi-erect; 5, horizontal; 7, semi-dropping	5	5	5
Inflorescence: type (2nd and 3rd truss)	IT	1, mainly uniparous; 2, intermediate; 3, mainly multiparous	1	1	1
Flower: fasciation (1st flower of inflorescences)	FF	1, absent; 9, present	1	1	1
Flower: pubescence of style	FPS	1, absent; 9, present	1	1	1
Flower: color	FC	1, yellow; 2, orange	1	1	1
Peduncle: abscission layer	PAL	1, absent; 9, present	9	9	9
Peduncle: length (from abscission layer to calyx)	PL	3, short; 5, medium; 7, long	3	3	3
Fruit: size	FrSz	1, very small; 3, small; 5, medium; 7, large; 9, very large	1	3	3
Fruit: ratio length/width	FrLW	1, very small; 3, small; 5, medium; 7, large; 9, very large	5	5	3
Fruit: shape in longitudinal section	FrSL	1, flattened; 2, slightly flattened; 3, circular; 4, rectangular; 5, cylindrical; 6, elliptic; 7, heart-shaped; 8, obovate; 9, ovate; 10, pear-shaped	4	3	2
Fruit: ribbing at peduncle end	FrRP	1, absent or very weak; 3, weak; 5, medium; 7, strong; 9, very strong	1	1	3
Fruit: cross section	FrCS	1, not round; 2, round	1	1	1
Fruit: depression at peduncle end	FrDP	1, absent or very weak; 3, weak; 5, medium; 7, strong; 9, very strong	1	3	5
Fruit: size of peduncle scar	FrPS	1, very small; 3, small; 5, medium; 7, large; 9, very large	1	1	5
Fruit: size of blossom scar	FrBS	1, very small; 3, small; 5, medium; 7, large; 9, very large	1	1	1
Fruit: shape at blossom end	FrSB	1, indented; 2 indented to flat; 3, flat; 4, flat to pointed; 5, pointed	3	3	3
Fruit: size of core in cross section (in relation to total diameter)	FrCS	1, very small; 3, small; 5, medium; 7, large; 9, very large	1	3	1
Fruit: thickness of pericarp	FrTP	3, thin; 5, medium; 7, thick	3	3	3
Fruit: number of locules	FrNL	1, only two; 2, two or three; 3, three or four; 4; four, five or six; 5, more than six	1	2	1
Fruit: green shoulder (before maturity)	FrGS	1, absent; 9, present	1	1	1
Fruit: intensity of green color (before maturity)	FrGC	3, light; 5, medium; 7, dark	3	3	3
Fruit: color at maturity	FrC	1, cream; 2, yellow; 3, orange; 4, pink; 5, red; 6, brownish	5	3	5
Fruit: color of flesh (at maturity)	FrCF	1, cream; 2, yellow; 3, orange; 4, pink; 5, red; 6, brownish	5	3	5
Fruit: firmness	FrF	1, very soft; 3, soft; 5, medium; 7, firm; 9, very firm	5	7	7
Time of flowering	TF	3, early; 5, medium; 7, late	3	3	3
Time of maturity	TM	1, very early; 3, early; 5, medium; 7, late; 9, very late	3	3	3

**Table 2:** Main effects of genotype and harvest time on color parameters (L*, a*, b*, h° and C) of tomato fruits.

Attributes	L*	a*	b*	h°	C
**Genotype**					
Manduria	36.4 b	27.8 b	31.2 b	48.2 b	41.8 b
Giallo di Crispiano	44.3 a	24.7 c	37.7 a	56.6 a	45.1 a
Regina	39.0 b	30.9 a	31.2 b	45.3 b	43.9 ab
**Harvest**					
First ^(A)^	38.7	27.6	33.7	50.7	43.7
Second ^(B)^	41.1	28.0	32.9	49.4	43.5
*Significance*					
Genotype (G)	**	**	**	**	*
Harvest (H)	**	ns	ns	ns	ns
G × H	ns	*	*	ns	*

^(A)^ 7th, 14th and 20th July 2016, respectively, for Manduria, Regina and Giallo di Crispiano; ^(B)^ 14th and 20th July and 4th August 2016, respectively, for Manduria, Regina and Giallo di Crispiano. *Significance*: ns = not significant; * and ** significant for *p* ≤ 0.05 and 0.01, respectively. Different letters indicate statistically significant differences at *p* = 0.05.

**Table 3:** Main effects of genotype and harvest time on weight, diameters, dry matter, total soluble solids (TSS), titratable acidity (TA) and TSS/TA ratio of tomato fruits.

Attributes	Fruit Weight (g)	Fruit Diameter (mm)		Dry Matter (g 100 g^−1^ FW)	TSS (°Brix)	TA (g 100 mL^−1^)	TSS/TA Ratio
		Polar	Equatorial				
**Genotype**							
Manduria	15.9 b	34.4 a	28.3 c	8.48	6.15	0.43 a	14.3 c
Giallo di Crispiano	19.2 b	29.0 c	34.0 b	8.82	6.55	0.38 b	17.4 b
Regina	29.7 a	32.6 b	39.5 a	8.87	6.53	0.34 c	19.3 a
**Harvest**							
First ^(A)^	24.7	33.3	35.5	8.43	6.11	0.38	16.4
Second ^(B)^	18.5	30.1	32.3	9.01	6.71	0.38	17.7
*Significance*							
Genotype (G)	**	**	**	ns	ns	***	***
Harvest (H)	*	ns	**	ns	ns	ns	Ns
G × H	ns	ns	ns	ns	ns	ns	Ns

^(A)^ 7th, 14th and 20th July 2016, respectively for Manduria, Regina and Giallo di Crispiano; ^(B)^ 14th and 20th July and 4th August 2016, respectively for Manduria, Regina and Giallo di Crispiano. FW = fresh weight. *Significance*: ns = not significant; *, ** and *** significant for *p* ≤ 0.05, 0.01 and 0.001, respectively. Different letters indicate statistically significant differences at *p* = 0.05.

**Table 4:** Main effects of genotype and harvest time on glucose and fructose content, glucose/fructose (Glu/Fru) ratio, sweetness index, ascorbic acid and total phenols content of tomato fruits.

Attributes	Glucose	Fructose	Glu/Fru Ratio	Swetness Index	Ascorbic Acid	Total Phenols
	(mg 100 g^−1^ FW)				(mg 100 g^−1^ FW)	
**Genotype**						
Manduria	2013 b	2316 c	0.86	7.34 c	58.2	72.9 c
Giallo di Crispiano	2140 b	2512 b	0.85	7.92 b	67.5	97.9 a
Regina	2332 a	2653 a	0.88	8.44 a	66.0	86.4 b
**Harvest**						
First ^(A)^	2099	2449	0.86	7.73	57.9	74.5
Second ^(B)^	2223	2538	0.87	8.06	69.9	97.0
*Significance*						
Genotype (G)	*	**	ns	**	ns	**
Harvest (H)	ns	ns	ns	ns	**	*
G × H	ns	ns	ns	ns	**	ns

^(A)^ 7th, 14th and 20th July 2016, respectively for Manduria, Regina and Giallo di Crispiano; ^(B)^ 14th and 20th July and 4th August 2016, respectively for Manduria, Regina and Giallo di Crispiano. *Significance*: ns = not significant; * and ** significant for *p* ≤ 0.05, and 0.01, respectively. Different letters indicate statistically significant differences at *p* = 0.05.

**Table 5:** Main effects of genotype and harvest time on carotenoids and tocopherols of tomato fruits.

Attributes	Lutein	α-Carotene	β-Carotene	Lycopene	α Tocopherol	β + γ Tocopherol
	(mg kg^−1^ FW)					
**Genotype**						
Manduria	2.81	0.42 b	5.39 b	19.59	10.59 b	2.63
Giallo di Crispiano	3.68	0.59 a	6.45 a	18.82	14.83 a	2.25
Regina	3.79	0.39 b	4.15 c	16.30	12.05 b	2.10
**Harvest**						
First ^(A)^	3.33	0.43	5.53	18.22	12.04	2.23
Second ^(B)^	3.53	0.50	5.13	18.27	12.93	2.43
*Significance*						
Genotype (G)	ns	*	**	ns	**	Ns
Harvest (H)	ns	**	ns	ns	ns	Ns
G × H	ns	*	ns	ns	ns	Ns

^(A)^ 7th, 14th and 20th July 2016, respectively for Manduria, Regina and Giallo di Crispiano; ^(B)^ 14th and 20th July and 4th August 2016, respectively for Manduria, Regina and Giallo di Crispiano. *Significance*: ns = not significant; * and ** significant for *p* ≤ 0.05, and 0.01, respectively. Different letters indicate statistically significant differences at *p* = 0.05.

**Table 6:** Main effects of genotype and harvest time on mineral content of tomato fruits.

Attributes	Ca	K	Mg	Na	B	Mn	Zn	Fe	Cu
	(mg kg^−1^ FW)								
**Genotype**									
Manduria	84.1	2955	128	75.9 a	1.02	0.64 a	1.88 b	2.73	5.32
Giallo di Crispiano	97.3	3178	121	38.7 c	1.05	0.61 a	4.22 a	2.87	6.10
Regina	94.5	2945	105	51.2 b	1.12	0.40 b	2.60 b	2.57	5.47
**Harvest**									
First ^(A)^	85.5	3009	110	51.7	1.08	0.52	2.61	2.94	5.08
Second ^(B)^	98.5	3043	126	55.8	1.05	0.59	3.53	2.51	6.18
*Significance*									
Genotype (G)	ns	ns	ns	***	ns	*	**	ns	ns
Harvest (H)	*	ns	*	ns	ns	ns	ns	*	ns
G × H	ns	ns	ns	*	ns	ns	ns	ns	ns

^(A)^ 7th, 14th and 20th July 2016, respectively for Manduria, Regina and Giallo di Crispiano; ^(B)^ 14th and 20th July and 4th August 2016, respectively for Manduria, Regina and Giallo di Crispiano. *Significance*: ns = not significant; *, ** and *** significant for *p* ≤ 0.05, 0.01 and 0.001, respectively. Different letters indicate statistically significant differences at *p* = 0.05.

**Table 7:** Average temperatures (min and max) and cumulative rain for each day decade during the growing cycle (from transplanting [14 April] to harvest [4 August]) of the three local varieties. Data retrieved from *Dati rete in telemisura, Protezione civile Puglia—Centro Funzionale Decentrato* [35]. Location of the weather station: 40°60′ N, 17°13′ E.

Day Decade	Temperature (°C)		Cumulated Rain (mm)
	min	max	
1	12.8	21.8	31
2	11.9	18.7	66
3	14.4	22.3	82
4	14.2	21.6	112
5	17.5	25.1	112
6	18.3	26.5	126
7	20.7	26.7	129
8	22.3	28.7	129
9	23.3	30.2	151
10	21.2	27.8	155
11	23.1	29.3	155

## Supplementary Materials

The following are available online at https://www.mdpi.com/2223-7747/8/8/273/s1, Table S1: Pearson correlation matrix for the physical and chemical observations analyzed in the Principal Component Analysis.
